# System-Level Identification of Heat Stress-Responsive Pathways in Human Saliva

**DOI:** 10.3390/ijms27167270

**Published:** 2026-08-14

**Authors:** Cassandra Lupita, Magda-Mihaela Luca, Anca-Cristina Perpelea, Iulia Muntean, Edida Maghet, Oana-Ramona Lobonț, Laura-Cristina Rusu

**Affiliations:** 1 Department of Oral Pathology, Multidisciplinary Center for Research, Evaluation, Diagnosis and Therapies in Oral Medicine, “Victor Babes” University of Medicine and Pharmacy Timisoara, Eftimie Murgu Square 2, 300041 Timisoara, Romania; cassandra.lupita@umft.ro (C.L.); iulia.sauciur@umft.ro (I.M.); edida.maghet@student.umft.ro (E.M.); laura.rusu@umft.ro (L.-C.R.); 2 Department of Pediatric Dentistry, Faculty of Dental Medicine, “Victor Babes” University of Medicine and Pharmacy Timisoara, Eftimie Murgu Square 2, 300041 Timisoara, Romania; 3 Department of Organization, Professional Legislation and Management of the Dental Office, Faculty of Dental Medicine, “Carol Davila” University of Medicine and Pharmacy, 17-23 Plevnei Street, 020021 Bucharest, Romania; 4 Finance, Business Information Systems and Modeling Department, Faculty of Economics and Business Administration, West University of Timisoara, 300115 Timisoara, Romania; oana.lobont@e-uvt.ro

**Keywords:** human saliva, heat stress, salivary proteome, systems biology, transcriptomic validation, oral biomarkers

## Abstract

Environmental heat stress disrupts cellular homeostasis through coordinated molecular responses involving protein quality control, oxidative stress regulation, inflammatory signaling, and water homeostasis. Although saliva plays an essential role in maintaining oral homeostasis, the molecular pathways underlying salivary adaptation to heat stress remain insufficiently characterized. We hypothesized that integrating the human salivary proteome with systems biology approaches would identify a reproducible heat-stress-responsive molecular signature. Human salivary proteomic datasets retrieved from the Human Salivary Proteome Wiki were integrated into a non-redundant dataset comprising 15,594 protein accessions corresponding to 2540 distinct proteins. Following deduplication, functional annotation, and biological curation, proteins associated with heat stress response, oxidative stress regulation, inflammatory signaling, water homeostasis, salivary secretion, and mucosal protection were assembled into a Salivary Climate Stress Panel (SCSP). Protein–protein interaction and functional enrichment analyses were performed, and the biological relevance of the identified proteins was independently evaluated using a human heat stress transcriptomic meta-analysis comprising 322 comparisons and the GEO dataset GDS3004. Functional filtering identified 2050 heat-stress-associated salivary proteins, from which a curated SCSP of 35 proteins was established. Network analysis identified HSP90AB1, HSP90AA1, and IL1B as the principal hub proteins linking heat stress, oxidative stress, inflammatory signaling, and water homeostasis pathways, while transcriptomic validation confirmed consistent activation of representative SCSP genes under heat stress conditions. These findings provide a reproducible systems-level framework for investigating heat-stress-responsive molecular pathways in the human salivary proteome and support future studies on salivary biomarkers of environmental heat exposure.

## 1. Introduction

Climate change is increasing the frequency and intensity of heat waves and other conditions that expose people to excessive heat [1,2,3]. In physiological terms, heat stress occurs when the body gains or produces more heat than it can dissipate while maintaining a stable internal temperature. The thermal load may come from the environment, such as high air temperature, humidity, and solar radiation, or from within the body, especially metabolic heat generated during physical activity, fever, or illness. When heat dissipation is insufficient, dehydration, osmotic imbalance, oxidative stress, inflammatory activation, and cellular injury may follow [4,5,6]. These effects have been widely studied in cardiovascular, respiratory, and metabolic systems [1,2,7], but their influence on saliva and oral homeostasis remains less well understood [8,9].

Saliva is a complex and highly dynamic biological fluid that reflects both local oral processes and systemic physiological conditions. Beyond its well-established functions in lubrication, digestion, buffering, remineralization, and antimicrobial defense, saliva contributes to the maintenance of oral homeostasis through immune regulation, tissue protection, and modulation of the oral microbiome [8,9,10,11]. Alterations in salivary flow rate or composition have been associated with xerostomia, dental caries, periodontal diseases, mucosal disorders, and systemic conditions, highlighting the importance of salivary gland function for oral and general health [9,12,13]. Owing to its non-invasive accessibility and rich molecular composition, saliva has increasingly been recognized as an attractive diagnostic medium for biomarker discovery and health monitoring. Advances in proteomics, metabolomics, transcriptomics, and microbiome research have further established saliva as a valuable source of information capable of reflecting physiological adaptation, disease progression, and environmental exposures [14,15,16].

The biological response also depends on the duration and intensity of exposure. Acute heat stress develops over minutes to hours and produces rapid responses, including sweating, fluid and electrolyte loss, osmotic changes, oxidative stress, and induction of heat shock proteins. Repeated or chronic exposure may lead to partial acclimation, but persistent dehydration and recurrent cellular stress may also alter inflammatory regulation, glandular function, and tissue homeostasis [6,17,18,19,20,21,22]. In salivary tissues, these processes can affect protein folding, reactive oxygen species (ROS) control, NF-κB-mediated inflammatory signaling, water transport, and secretion. Thus, environmental heat and internally generated metabolic heat may activate overlapping pathways, although their magnitude and clinical effects depend on the exposure context.

Several studies have demonstrated that dehydration and heat exposure alter salivary flow rate, viscosity, electrolyte composition, and stress-associated biomarkers, suggesting that saliva may serve as a sensitive indicator of physiological adaptation to environmental heat stress [23,24,25]. Furthermore, proteins involved in water transport, including aquaporins, have been recognized as key regulators of salivary gland function and fluid secretion, highlighting the relevance of water-homeostasis pathways in the context of thermal adaptation [26,27]. Despite these observations, current knowledge remains fragmented, with most studies focusing on individual biomarkers or isolated physiological responses rather than examining the integrated molecular architecture of the salivary proteome under heat stress conditions.

High-throughput proteomics has shown that saliva contains thousands of proteins involved in immune defense, tissue repair, antimicrobial activity, signaling, metabolism, and stress adaptation [28,29,30,31]. Repositories such as the Human Salivary Proteome Wiki make these data available for systematic analysis [30,32]. They therefore allow salivary proteins to be studied as connected biological pathways rather than as isolated markers. Public transcriptomic datasets can provide an additional, independent test of whether genes corresponding to selected salivary proteins respond to heat under controlled conditions.

Salivary proteins do not act independently. They form networks that contribute to cellular adaptation, immune responses, oxidative balance, and fluid homeostasis [33,34]. Systems biology examines these connections and can identify highly connected proteins, functional modules, and shared pathways. Applied to heat stress, this approach can reveal coordinated responses that would be missed by examining one biomarker at a time.

Despite growing interest in heat stress physiology and the expanding knowledge of the human salivary proteome, no study has systematically investigated heat-stress-responsive molecular pathways within saliva using an integrated systems biology framework. Existing research has primarily focused on individual biomarkers, isolated physiological responses, or specific disease-related mechanisms, leaving the broader molecular organization of salivary adaptation to environmental heat stress largely unexplored. To our knowledge, this is the first study integrating the complete human salivary proteome with protein–protein interaction network analysis, Gene Ontology functional annotation, and independent transcriptomic validation to identify a systems-level heat-stress-responsive molecular network within the human salivary proteome. Addressing this knowledge gap is particularly relevant in the context of increasing environmental heat exposure, where coordinated molecular responses may contribute to the maintenance of oral homeostasis under thermal stress.

We hypothesized that proteins involved in heat-stress response, oxidative-stress regulation, inflammatory signaling, and water-homeostasis mechanisms form interconnected molecular networks within the human salivary proteome. Therefore, the aim of the present study was to identify and characterize a Salivary Climate Stress Panel (SCSP) by integrating large-scale human salivary proteomic datasets with protein–protein interaction network analysis, Gene Ontology functional annotation, and independent transcriptomic validation. Salivary proteins associated with predefined heat-stress-related biological processes were systematically identified through functional annotation and biological curation, organized into functional modules, and analyzed using a systems biology framework. The resulting molecular network was subsequently evaluated through independent transcriptomic validation to assess its biological relevance under heat stress conditions. Collectively, this work provides a reproducible systems-level framework for future investigations of salivary biomarkers associated with environmental heat exposure and advances our understanding of the molecular mechanisms underlying salivary adaptation to thermal stress.

## 2. Results

### 2.1. Construction of the Salivary Climate Stress Panel (SCSP)

Application of the functional filtering strategy to the integrated salivary proteome dataset resulted in the identification of 2050 proteins meeting the predefined heat-stress-related functional filtering criteria. These proteins were assigned to one or more of the six predefined functional categories, including heat stress response, oxidative stress regulation, inflammatory signaling, water homeostasis, salivary secretion, and mucosal protection.

Among the identified proteins, the largest functional category corresponded to inflammation and immune-response pathways (747 proteins; 36.4%), followed by proteins involved in mucosal protection and antimicrobial defense (482 proteins; 23.5%). Heat-stress-related proteins accounted for 321 candidates (15.7%), whereas oxidative stress and reactive oxygen species (ROS)-associated proteins represented 272 candidates (13.3%). Proteins involved in water transport, dehydration response, and osmotic regulation accounted for 111 candidates (5.4%). A summary of the functional filtering process is presented in Table 1.

**Figure 1:**
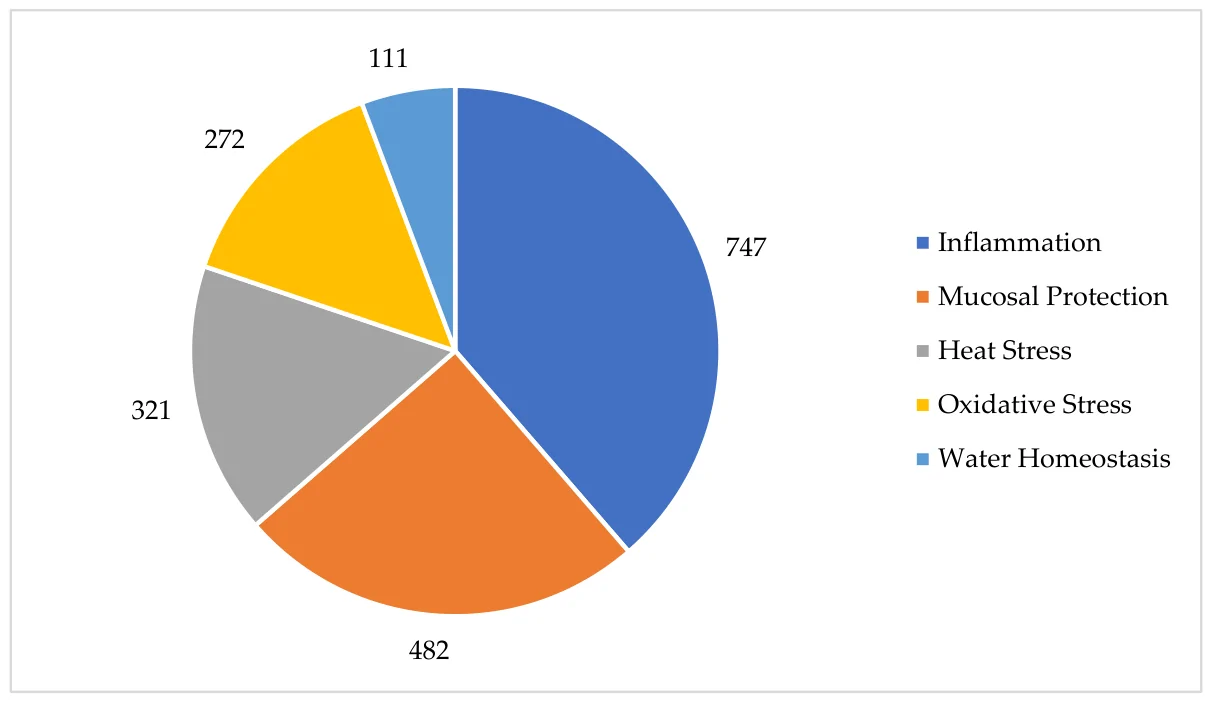
Distribution of heat-stress-related protein categories identified during functional filtering of the human salivary proteome. Inflammatory and immune-response proteins constituted the largest functional category, followed by mucosal protection, heat stress, oxidative stress, and water-homeostasis-associated proteins.

The distribution of proteins among the principal heat-stress-related functional categories is illustrated in Figure 1. Inflammatory and immune-response proteins represented the largest category, followed by mucosal protection proteins and heat-stress-associated proteins. Together, these categories accounted for the majority of proteins retained after functional filtering.

Following computational filtering, the 2050 candidates were subjected to expert-driven biological refinement using the prespecified criteria detailed in Section 4.4. Briefly, a protein had to be present in the integrated salivary proteome, have annotation-supported relevance to at least one of the six functional categories, and contribute a distinct regulatory or effector role. Canonical representatives with direct relevance to heat response or salivary physiology were prioritized, whereas proteins with substantially overlapping functions were reduced to limit redundancy. This purposive procedure produced the proposed SCSP of 35 proteins distributed across six categories (Table 2). The number 35 was not derived from a statistical cut-off; it reflects a focused, biologically interpretable panel for pathway-level analysis.

### 2.2. Protein–Protein Interaction Network Analysis

To investigate the molecular relationships among proteins included in the Salivary Climate Stress Panel (SCSP), a protein–protein interaction (PPI) network was generated using the STRING database. STRING mapped the 35 SCSP input identifiers to 34 network nodes and identified 72 protein–protein associations at the prespecified confidence threshold of 0.700. The network contained substantially more edges than the 13 expected by chance (PPI enrichment *p* < 1.0 × 10^−16^), with an average node degree of 4.24 and an average local clustering coefficient of 0.618. These statistics support a non-random and biologically coherent organization of the mapped proteins.

**Figure 2:**
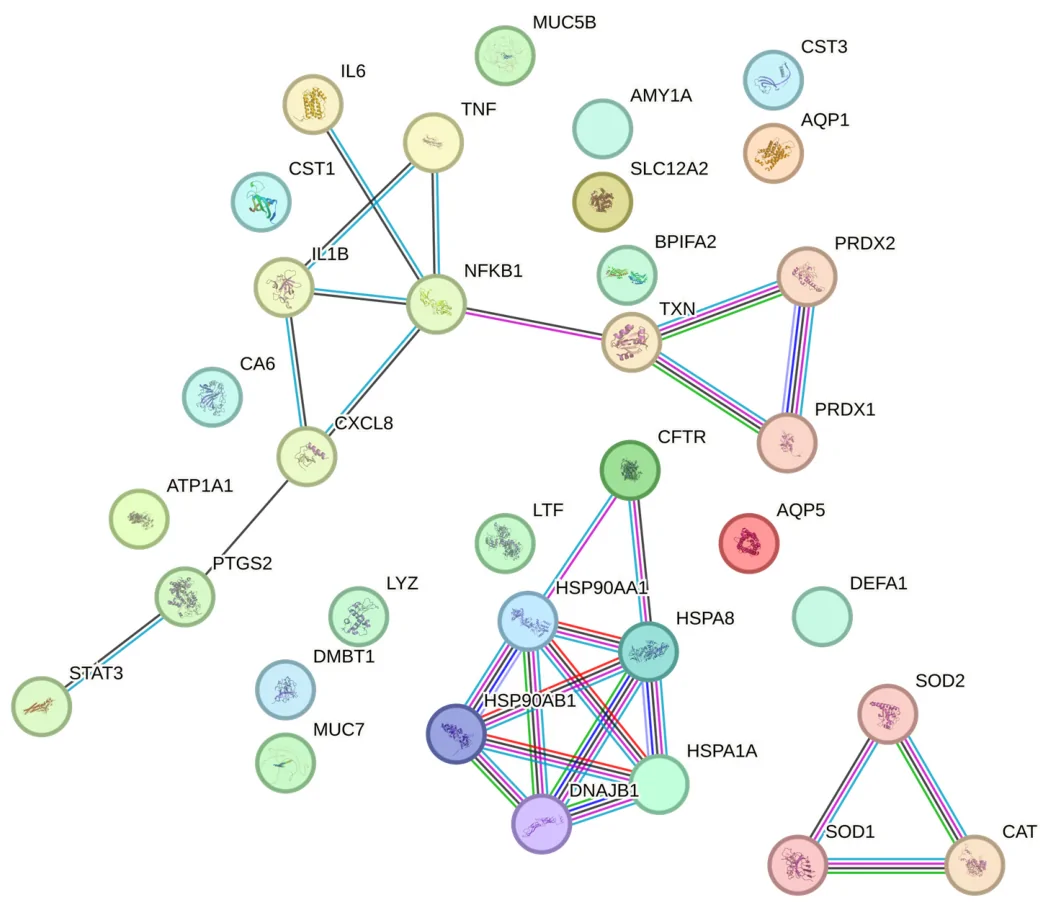
Protein–protein interaction network of the proposed Salivary Climate Stress Panel (SCSP). The 35 input identifiers were analyzed in STRING version 12.0 (Homo sapiens, confidence score ≥ 0.700) and mapped to 34 network nodes. Four major functional modules corresponding to heat-stress response, inflammatory signaling, oxidative-stress regulation, and water-homeostasis mechanisms can be identified. The heat-stress module constitutes the central and most densely connected region of the network.

Network topology analysis revealed a structured interaction landscape characterized by four major functional modules corresponding to heat-stress response, inflammatory signaling, oxidative-stress regulation, and water homeostasis (Figure 2). The heat-stress module formed the most densely connected region of the network and was centered on HSPA1A, HSPA8, HSP90AA1, HSP90AB1, and DNAJB1. The inflammatory module included NFKB1, IL6, TNF, IL1B, CXCL8, STAT3, and PTGS2, whereas the oxidative-stress module comprised TXN, SOD1, SOD2, CAT, PRDX1, PRDX2, and GPX1. Proteins involved in water transport and salivary gland physiology, including AQP1, AQP5, CFTR, ATP1A1, and SLC12A2, exhibited lower levels of connectivity.

The general network characteristics are summarized in Table 3.

To identify key regulatory proteins within the network, node degree analysis was performed using the same STRING output. The highest-ranked proteins are presented in Table 4. HSP90AB1 and HSP90AA1 showed the greatest connectivity (degrees 12 and 11, respectively), followed by IL1B (degree 9). IL6, NFKB1, SOD1, and STAT3 each had a degree of 8. These findings position molecular chaperones at the core of the SCSP while also demonstrating dense links with inflammatory and redox-regulatory proteins.

### 2.3. Functional Enrichment Analysis

Gene Ontology (GO)-based functional annotation was performed to characterize the principal biological processes represented within the Salivary Climate Stress Panel (SCSP). The analysis identified enrichment of biological processes related to water transport, water homeostasis, osmoregulation, salivary secretion, oxidative stress response, and physiological homeostasis.

Among the proteins included in the SCSP, AQP1 and AQP5 were associated with the largest number of annotated Gene Ontology biological processes related to water transport and salivary gland physiology.

AQP1 showed the broadest annotation pattern and was linked to water transport, water and cellular homeostasis, osmotic and oxidative stress responses, regulation of saliva secretion, body fluid secretion, and chemical homeostasis. AQP5 was linked to water transport, stress and osmotic responses, saliva secretion, and body fluid secretion.

The STRING Gene Ontology enrichment analysis identified significant convergence across osmotic, thermal, oxidative, inflammatory, protein-folding, and antimicrobial response processes. Table 5 reports representative biological processes together with the observed gene count, Benjamini–Hochberg’s FDR-adjusted *p*-value, and matching SCSP proteins.

### 2.4. Identification of the Heat-Stress-Responsive Salivary Signature

Combining the PPI network with functional annotation showed that the SCSP proteins could be organized into related biological modules. The panel should therefore be viewed as a pathway-level model of salivary responses relevant to heat stress, rather than as a set of independently validated biomarkers.

**Figure 3:**
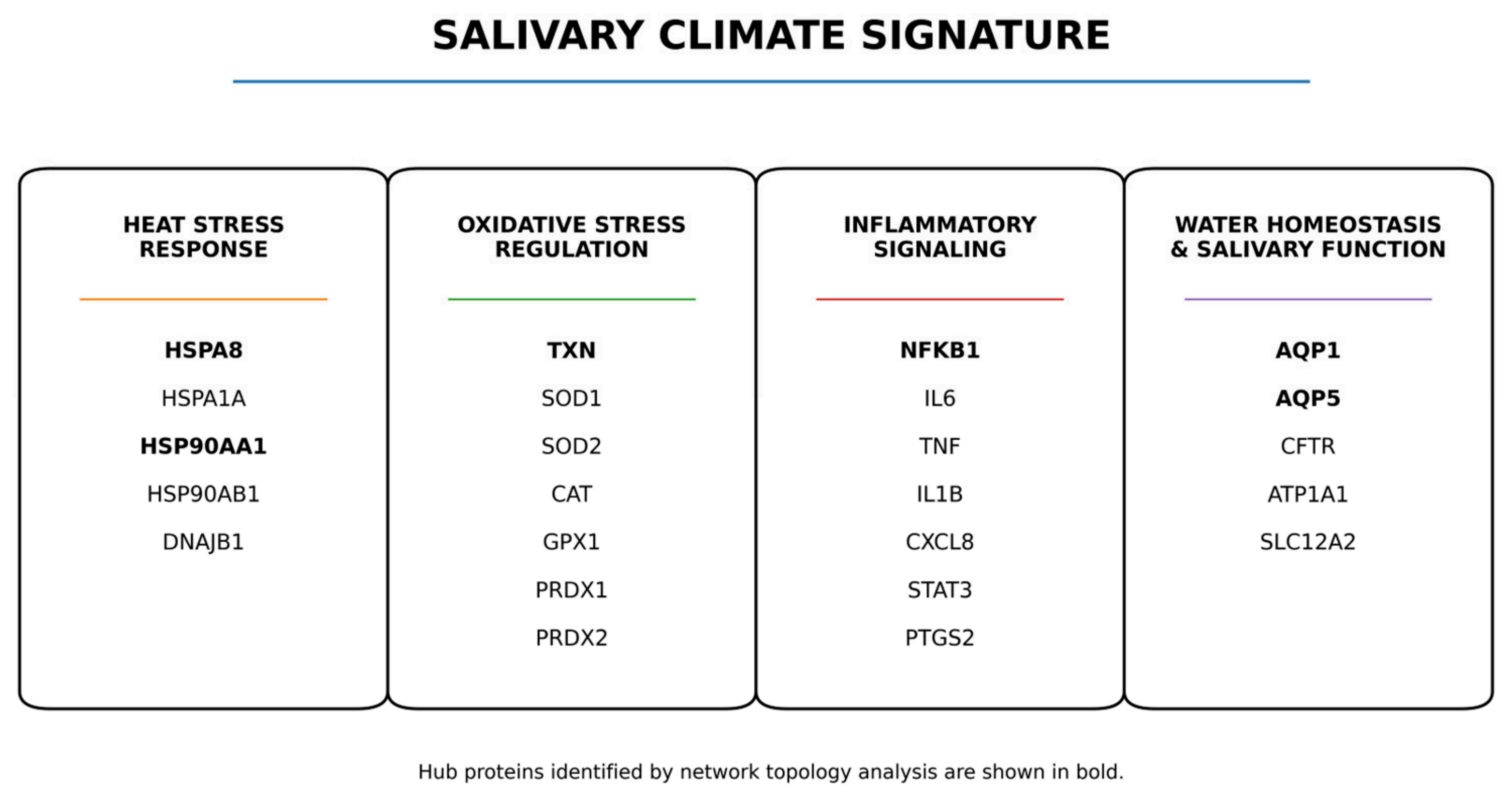
Functional organization of the proposed Heat-Stress-Responsive Salivary Signature. The identified proteins clustered into four interconnected biological modules corresponding to heat stress response, oxidative stress regulation, inflammatory signaling, and water homeostasis. Together, these modules define the systems-level architecture of the proposed Heat-Stress-Responsive Salivary Signature.

The obtained signature consisted of four main biological modules associated with heat stress, oxidative stress, inflammation, and water homeostasis (Figure 3). The heat stress module was primarily composed of representatives of the heat shock protein family, such as HSPA1A, HSPA8, HSP90AA1, HSP90AB1, and DNAJB1. These proteins took central positions in the interaction network. The oxidative stress module contained SOD1, SOD2, CAT, TXN, PRDX1, PRDX2, and GPX1, while the inflammatory module was composed of NFKB1, IL6, TNF, IL1B, CXCL8, STAT3, and PTGS2. Proteins responsible for water transport and salivary gland function (e.g., AQP1, AQP5, CFTR, ATP1A1, and SLC12A2) formed the fourth biological module.

The modules were linked through protein–protein interactions and shared functional pathways. This organization suggests that salivary adaptation may involve coordinated stress, redox, immune, and homeostatic responses rather than the action of a single protein.

Together, these results define a proposed Heat-Stress-Responsive Salivary Signature composed of four linked modules. This signature is a hypothesis-generating framework for future studies of salivary responses to heat exposure.

### 2.5. Proposed Heat Stress Response Axis

**Figure 4:**
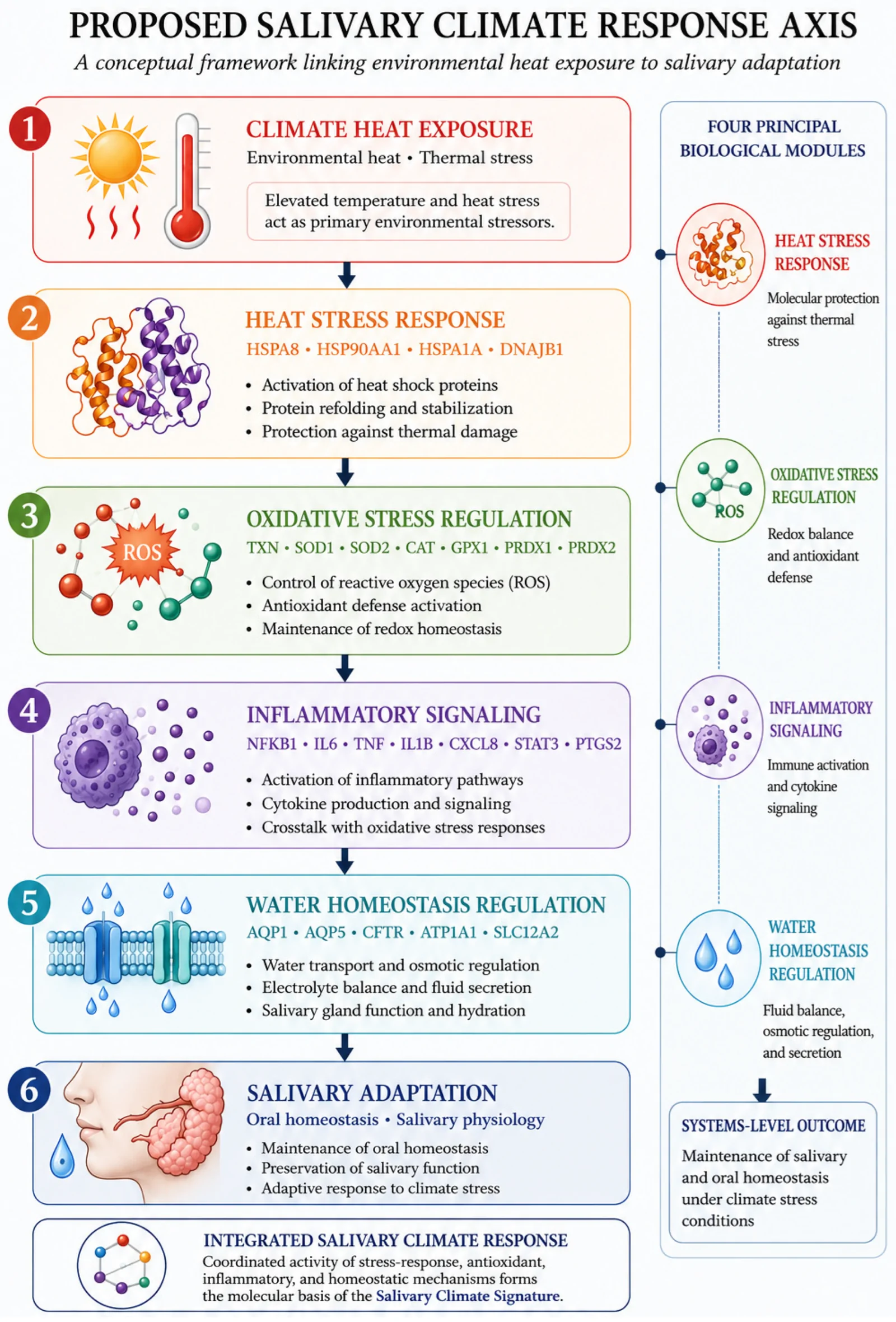
Proposed Heat Stress Response Axis.

Integration of the results obtained from functional protein filtering, protein–protein interaction network analysis, Gene Ontology functional annotation, and independent transcriptomic validation enabled the development of a conceptual framework termed the Salivary Heat Stress Response Axis (Figure 4). The framework summarizes the principal molecular processes represented within the Salivary Climate Stress Panel (SCSP) and their organization into interconnected biological modules associated with salivary adaptation to environmental heat stress.

The proposed Salivary Heat Stress Response Axis is organized into four principal biological modules identified in the present study: heat stress response, oxidative stress regulation, inflammatory signaling, and water homeostasis regulation. The heat stress module comprises the molecular chaperones HSPA1A, HSPA8, HSP90AA1, HSP90AB1, and DNAJB1, which formed the central interaction cluster within the protein–protein interaction network. The oxidative stress module includes TXN, SOD1, SOD2, CAT, GPX1, PRDX1, and PRDX2, whereas the inflammatory signaling module contains NFKB1, IL6, TNF, IL1B, CXCL8, STAT3, and PTGS2. Proteins involved in water transport and salivary gland physiology, including AQP1, AQP5, CFTR, ATP1A1, and SLC12A2, constitute the water homeostasis module and are associated with biological processes related to water transport, osmotic regulation, and salivary secretion.

Within the proposed framework, these four biological modules converge toward maintenance of salivary gland function and oral homeostasis, representing the systems-level organization of the molecular pathways identified through the integrated proteomic, network, functional annotation, and transcriptomic analyses. The Salivary Heat Stress Response Axis therefore provides a conceptual representation of the molecular architecture identified in the present study and serves as a framework for future experimental investigation of heat-stress-responsive salivary adaptation.

Conceptual framework summarizing the principal molecular pathways represented within the Salivary Climate Stress Panel (SCSP). The model integrates the four major biological modules identified through protein–protein interaction network analysis, Gene Ontology functional annotation, and independent transcriptomic validation, including heat stress response, oxidative stress regulation, inflammatory signaling, and water homeostasis regulation. The proposed axis represents a conceptual framework derived from the integrated analyses performed in this study and should not be interpreted as evidence of a causal or temporal sequence of biological events.

### 2.6. External Validation of the Heat-Stress-Responsive Salivary Signature

Independent validation using public heat-stress transcriptomic datasets provided additional support for the overlap between proteins included in the proposed Salivary Climate Stress Panel (SCSP) and canonical heat-responsive molecular pathways.

Table 6 presents the HN scores obtained for representative SCSP proteins identified within the published human heat-stress transcriptomic meta-analysis [35].

**Figure 5:**
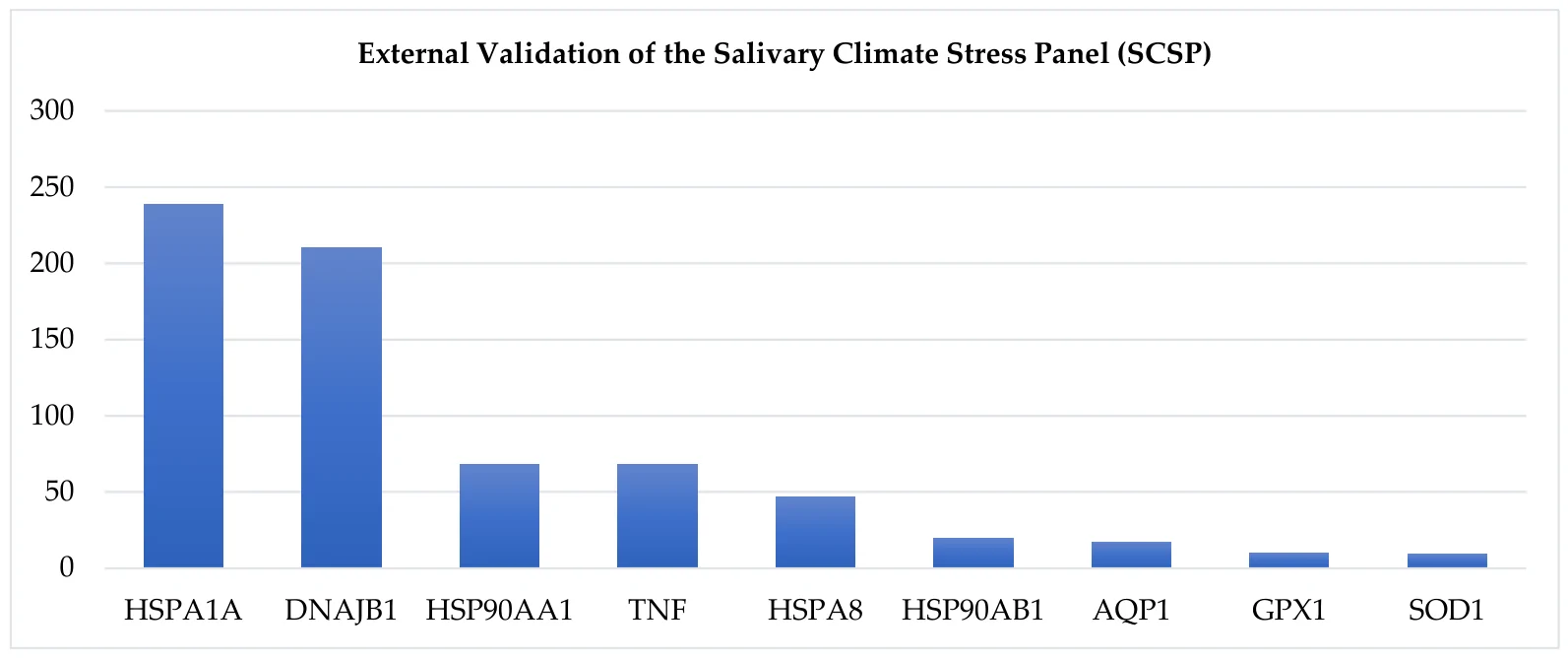
Validation of key Salivary Climate Stress Panel (SCSP) genes using a human heat-stress transcriptomic meta-analysis comprising 322 independent heat-stress versus control comparisons.

To facilitate visualization of the external validation results, the HN scores of the principal SCSP genes identified within the published human heat-stress transcriptomic meta-analysis are presented in Figure 5 [35].

The biological relevance of the proposed signature was also examined in GDS3004, a transcriptomic dataset of human epithelial cells exposed to temperatures from 37 °C to 43 °C. Representative genes from the main SCSP modules (DNAJB1, HSP90AA1, HSPA8, TNF, and AQP1) were selected. Row-wise z-score normalization was used to compare temperature-related expression patterns across genes with different baseline levels. This analysis tested whether the selected genes responded to controlled heating; it was not considered direct evidence of changes in salivary protein abundance.

**Figure 6:**
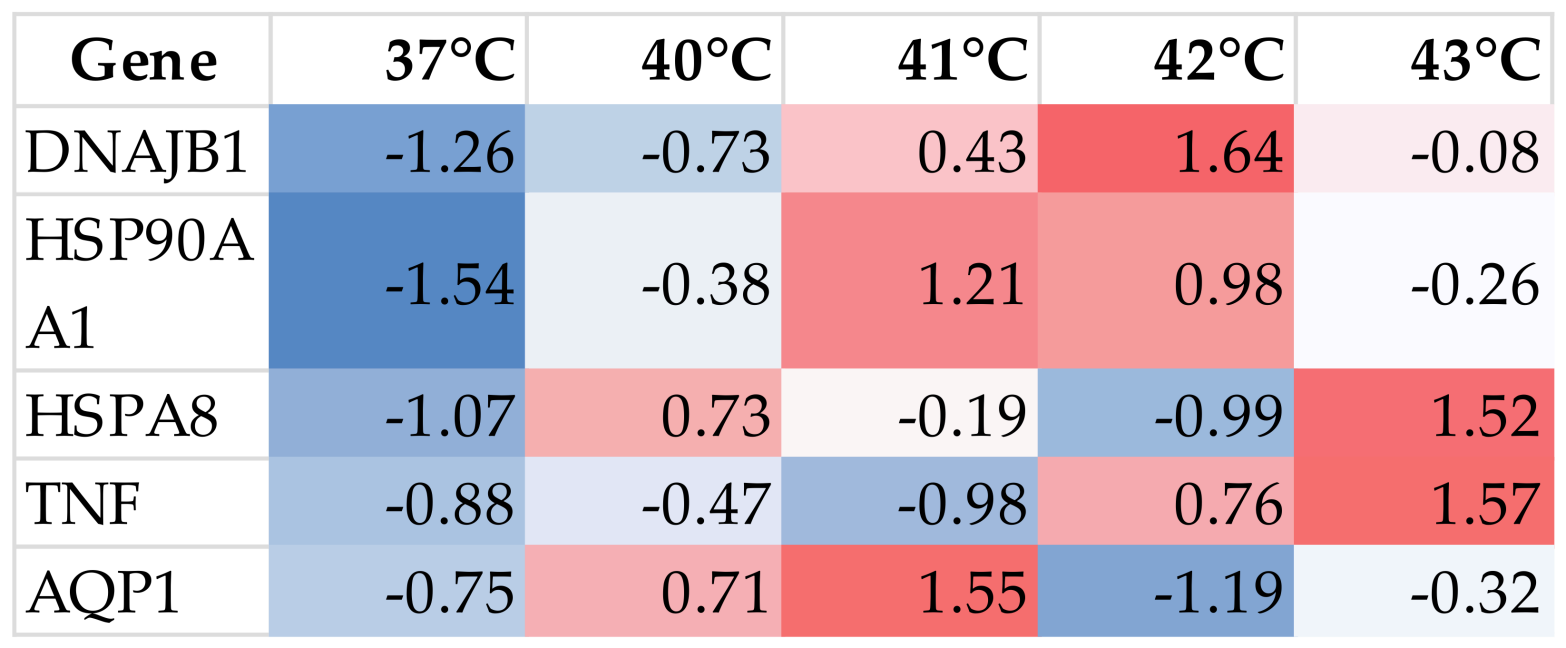
Heatmap of representative SCSP genes across increasing heat-stress conditions in the GDS3004 dataset.

The resulting heatmap revealed distinct temperature-responsive expression profiles among the selected SCSP genes (Figure 6). The strongest responses were observed for DNAJB1 and HSP90AA1, both of which demonstrated marked upregulation at elevated temperatures, particularly between 41 °C and 42 °C. These findings are consistent with their established roles as molecular chaperones involved in cellular protection against thermal stress and support their identification as central hub proteins within the heat-stress module of the proposed Heat-Stress-Responsive Salivary Signature.

Comparison of SCSP genes with the human Heat-Stress–Non-treatment (HN) meta-analysis database revealed that several of the principal hub proteins identified through network analysis were consistently upregulated across 322 independent human heat-stress transcriptomic experiments. The strongest responses were observed for HSPA1A (HN score = 239) and DNAJB1 (HN score = 210), both of which belong to the heat shock protein response system and represent key regulators of cellular adaptation to thermal stress.

Additional SCSP hub proteins, including HSP90AA1 (HN score = 68), HSPA8 (HN score = 47), TNF (HN score = 68), and AQP1 (HN score = 17), also demonstrated positive HN scores, indicating recurrent activation under heat-stress conditions. These findings provide independent support for the biological relevance of the heat-stress, inflammatory, and water-homeostasis modules identified through salivary proteome network analysis.

The GDS3004 validation further demonstrated temperature-dependent expression changes among representative SCSP genes. DNAJB1 and HSP90AA1 exhibited the most pronounced responses, showing progressive induction under increasing thermal stress conditions. TNF displayed a moderate increase at higher temperatures, whereas AQP1 exhibited a more variable profile, consistent with its role as a downstream physiological effector involved in water-homeostasis regulation rather than an immediate heat-stress response gene.

Taken together, the analyses show agreement at the pathway level between the salivary protein network and gene responses reported in independent heat-stress datasets. This agreement supports the biological plausibility of the proposed signature, but it does not establish that the corresponding proteins change in saliva after heat exposure. The SCSP should therefore be used as a hypothesis-generating framework for future salivary proteomic studies.

## 3. Discussion

### 3.1. Principal Findings

To our knowledge, this is the first study to examine heat-stress-related pathways across the human salivary proteome using protein interaction networks, Gene Ontology annotation, and independent transcriptomic evidence. Unlike studies centered on single salivary markers, this approach describes how several stress-related pathways may operate together.

The study produced four main findings. First, functional filtering identified 2050 salivary proteins associated with at least one predefined stress-related category. Second, the final panel formed linked modules related to heat shock, oxidative stress, inflammation, and water homeostasis. Third, Gene Ontology annotation highlighted water transport, salivary secretion, and homeostatic regulation, particularly for AQP1 and AQP5. Fourth, these results were integrated into the proposed Salivary Heat Stress Response Axis, a conceptual model of the pathways represented in the SCSP.

Together, these findings expand the current understanding of saliva as a dynamic biological system involved in physiological adaptation to environmental heat stress and provide a foundation for future experimental studies investigating heat-stress-responsive salivary biomarkers and their potential applications in oral biology and environmental health.

### 3.2. Biological Interpretation of the Salivary Climate Stress Panel

The systems-level organization identified in the present study is broadly consistent with current knowledge regarding the molecular response to environmental heat stress. Previous experimental studies have shown that thermal stress simultaneously activates molecular chaperones, antioxidant defense systems, inflammatory signaling pathways, and mechanisms regulating cellular and fluid homeostasis. Rather than identifying individual biomarkers, the present analysis demonstrates that these biological processes are represented as interconnected functional modules within the human salivary proteome, providing a systems-level perspective of salivary adaptation to environmental heat stress.

One of the most notable findings was the predominance of inflammatory and immune-response proteins, which represented the largest functional category identified during functional filtering. This observation is biologically plausible because inflammatory signaling integrates multiple stress-responsive pathways activated during thermal stress, including cytokine production, oxidative stress responses, epithelial defense, and immune regulation. Consequently, proteins involved in inflammatory signaling participate in a wide range of physiological processes and are represented across numerous biological databases, increasing their likelihood of being retained during systems-level protein selection.

Another important observation was the prominent representation of AQP1 throughout the Gene Ontology functional annotation. Unlike most proteins included in the SCSP, AQP1 was associated with numerous biological processes related to water transport, osmotic regulation, fluid homeostasis, body fluid secretion, and cellular homeostasis. This broad functional annotation reflects the central physiological role of aquaporins in maintaining water balance rather than increased biological importance relative to other proteins. Aquaporins are well-established regulators of salivary gland fluid secretion, and both AQP1 and AQP5 have been extensively implicated in salivary gland physiology and responses to osmotic stress, supporting their central position within the proposed framework.

The transcriptomic analyses provide complementary support for the biological plausibility of the SCSP. They show that several genes corresponding to proteins in the panel respond to heat in independent experiments. However, these experiments used non-salivary transcriptomic models and did not measure salivary proteins. Moreover, mRNA abundance may differ from protein abundance because of post-transcriptional regulation, protein turnover, secretion dynamics, and post-translational modification. The transcriptomic findings therefore support the selected pathways, but they do not directly validate protein expression in saliva.

Together, these findings indicate that the Salivary Climate Stress Panel captures multiple complementary aspects of the cellular response to environmental heat stress, integrating molecular chaperones, antioxidant defense, inflammatory signaling, and water homeostasis into a coherent systems-level framework that extends beyond individual biomarker-based approaches.

### 3.3. Heat Stress as the Central Regulatory Module of the Heat-Stress-Responsive Salivary Signature

The predominance of heat shock proteins within the SCSP suggests that cellular stress-response mechanisms occupy a central position within the proposed Heat-Stress-Responsive Salivary Signature. HSPA8, HSP90AA1, HSPA1A, HSP90AB1, and DNAJB1 represented five of the six highest-ranked hub proteins, indicating that molecular chaperone systems may coordinate multiple adaptive pathways simultaneously.

Heat shock proteins are among the earliest cellular responses to thermal stress and play essential roles in maintaining proteome integrity and preventing stress-induced protein aggregation. The central network positions of HSPA8 and HSP90AA1 suggest that constitutive and inducible chaperone systems may act as major regulators of heat-stress-responsive adaptation.

Interactions between heat shock proteins, oxidative-stress regulators, and inflammatory mediators further indicate that thermal stress responses may function as upstream coordinators of broader adaptive processes. Collectively, these findings support the concept that molecular chaperones constitute the core regulatory module of the proposed Heat-Stress-Responsive Salivary Signature.

### 3.4. Oxidative Stress and Inflammatory Crosstalk in Climate-Responsive Salivary Adaptation

The close integration between oxidative-stress and inflammatory pathways suggests that these processes function as complementary components of climate-responsive adaptation. The oxidative module, centered on TXN, SOD1, SOD2, CAT, GPX1, PRDX1, and PRDX2, was extensively connected with inflammatory mediators including NFKB1, IL6, TNF, IL1B, CXCL8, STAT3, and PTGS2.

The central network position of TXN suggests a potential bridging role between redox regulation and inflammatory activation, whereas the prominence of NFKB1 highlights the importance of immune signalling within thermal stress adaptation.

These findings are consistent with the well-established bidirectional relationship between oxidative stress and inflammation and suggest that redox and immune pathways represent critical intermediaries linking environmental heat exposure to downstream physiological responses.

### 3.5. Water Homeostasis as the Final Physiological Effector of Heat Stress Adaptation

Gene Ontology functional annotation identified water transport and homeostasis among the most prominently represented biological processes within the proposed Salivary Climate Stress Panel (SCSP). AQP1 and AQP5 were associated with multiple biological processes related to water transport, osmotic regulation, body fluid secretion, and salivary secretion, highlighting the importance of water homeostasis within the proposed molecular framework.

Unlike molecular chaperones and inflammatory mediators, aquaporins represent downstream physiological effectors that directly regulate fluid movement across salivary gland tissues. Among the thirteen mammalian aquaporins identified to date, AQP5 is considered the principal water channel in acinar cells of the major salivary glands, where it mediates transcellular water transport required for primary saliva secretion. AQP1 is predominantly expressed in vascular endothelial and myoepithelial cells and contributes to water exchange between the circulation and glandular tissue, thereby supporting salivary fluid production. Experimental studies have demonstrated that altered AQP5 expression is associated with impaired salivary secretion in conditions such as Sjögren’s syndrome, radiation-induced xerostomia, and other disorders affecting salivary gland function, emphasizing the physiological importance of aquaporin-mediated water transport.

These observations are consistent with the proposed Salivary Heat Stress Response Axis, in which molecular stress-response pathways converge toward biological processes regulating water homeostasis and salivary secretion. Within this framework, alterations in aquaporin-associated pathways may represent an important molecular link between environmental heat stress, hydration status, and maintenance of oral homeostasis.

### 3.6. Potential Relevance to Oral Pathology

The mucosal-protection component of the SCSP provides a clinically relevant link between heat exposure, salivary function, and oral disease susceptibility. MUC5B and MUC7 contribute to lubrication, formation of the protective salivary film, microbial clearance, and maintenance of mucosal surface integrity, while lactoferrin (LTF) supports antimicrobial defense and modulation of inflammatory activity [8,9,10,11]. Reduced salivary flow during heat exposure could increase the concentration of some salivary components while decreasing their delivery and distribution across oral surfaces. Therefore, measured concentration and effective protective function should not be assumed to change in parallel.

If heat-related dehydration is accompanied by lower salivary flow or altered mucin secretion, impaired lubrication and a less stable mucosal barrier could contribute to dry-mouth symptoms, friction-related discomfort, and greater susceptibility to mucosal irritation. Changes in MUC5B or MUC7 could also modify microbial adhesion and clearance. However, the present study did not measure flow rate, xerostomia, mucosal lesions, or protein abundance, so these mechanisms remain testable clinical hypotheses rather than demonstrated consequences of heat exposure.

Periodontal relevance may arise from the combined mucosal and inflammatory modules. Lower salivary clearance and altered antimicrobial protection could favor plaque retention or ecological imbalance, while changes in LTF and inflammatory mediators such as IL6, TNF, IL1B, CXCL8, and NFKB1 may reflect or influence host inflammatory responses. Because these proteins are not specific to heat or periodontal disease, future studies should assess them together with periodontal indices, oral hygiene, microbiome composition, hydration, medication use, and other potential confounders. This combined evaluation is necessary to determine whether the SCSP identifies heat-related oral vulnerability rather than nonspecific inflammation.

### 3.7. Implications for Heat-Stress-Responsive Salivary Biomarkers

The proposed Heat-Stress-Responsive Salivary Signature provides a conceptual framework for identifying heat-stress-responsive salivary biomarkers. By integrating proteins involved in stress response, redox regulation, immune signaling, and water homeostasis, the SCSP captures the multifactorial nature of environmental adaptation more effectively than approaches focused on individual biomarkers.

Proteins representing different stages of the proposed adaptive cascade may have distinct translational relevance. Heat shock proteins may reflect early thermal stress responses, antioxidant proteins may indicate redox adaptation, inflammatory mediators may reflect downstream immune activation, and aquaporins may provide information regarding physiological adaptation at the level of salivary gland function.

Beyond oral biology, the proposed framework may have broader applications in environmental and occupational health. Salivary biomarkers associated with heat stress adaptation could potentially support physiological monitoring of individuals chronically exposed to elevated environmental temperatures, including outdoor workers, athletes, military personnel, and other populations at increased risk of heat-related illness. In addition, non-invasive saliva-based assessment may contribute to monitoring hydration status, evaluating physiological adaptation during prolonged heat exposure, and identifying individuals requiring preventive interventions. Although these potential applications require prospective clinical validation, they illustrate the translational value of integrating salivary proteomics with systems biology approaches.

Although the present study should be regarded as hypothesis-generating, the identified proteins represent candidates for targeted validation in future studies of salivary physiology and oral health. Such studies should examine whether changes in mucins, lactoferrin, inflammatory mediators, and water-homeostasis proteins occur together with reduced salivary flow, xerostomia symptoms, periodontal inflammation, or mucosal lesions during heat exposure.

### 3.8. Strengths and Limitations

The present study possesses several strengths that support the relevance of its findings. First, to our knowledge, it represents the first systematic systems-level investigation of heat-stress-responsive molecular pathways within the human salivary proteome using an integrated systems biology approach. By combining large-scale salivary proteomic resources with protein–protein interaction network analysis, Gene Ontology functional annotation, and independent transcriptomic validation, the study provides a comprehensive overview of biological mechanisms potentially involved in salivary adaptation to environmental heat stress.

A second strength lies in the integration of multiple complementary analytical approaches. Functional filtering, protein–protein interaction network analysis, Gene Ontology functional annotation, and transcriptomic validation enabled the identification of biologically coherent molecular modules rather than isolated proteins, leading to the development of the Salivary Climate Stress Panel (SCSP) and the proposed Salivary Heat Stress Response Axis. The incorporation of independent heat stress transcriptomic datasets provided complementary evidence supporting the biological relevance of the identified molecular pathways.

Another important strength is the translational potential of saliva as a biological medium. Saliva collection is non-invasive, inexpensive, and suitable for repeated measurements, making it an attractive source of biomarkers for large-scale environmental, occupational, and public health investigations. Consequently, the proteins identified in the present study represent promising candidates for future studies evaluating physiological responses to environmental heat exposure.

Despite these strengths, several limitations should be acknowledged. First, the study relied primarily on publicly available proteomic repositories and bioinformatic resources and did not include direct experimental validation in human subjects. Consequently, the proposed molecular framework should be interpreted as biologically plausible rather than experimentally confirmed.

Second, the resulting proteomic dataset is inherently influenced by publication bias, differences in experimental methodologies, and variability in protein identification among the original studies included in the Human Salivary Proteome Wiki. Furthermore, the selection of proteins included in the Salivary Climate Stress Panel involved knowledge-based biological curation according to predefined functional criteria. Although this approach improved biological interpretability, a certain degree of subjective selection cannot be completely excluded.

Third, the protein–protein interaction analysis was performed using the STRING database, which integrates experimentally validated as well as predicted protein interactions. Therefore, the identified interaction network reflects the current state of biological knowledge rather than direct experimental evidence of protein interactions occurring specifically within human saliva under environmental heat stress conditions.

Fourth, the analyses performed do not provide quantitative information regarding changes in protein abundance under specific environmental conditions. Identification of proteins associated with heat stress response, oxidative stress regulation, inflammatory signaling, and water homeostasis indicates potential biological involvement but does not demonstrate differential protein expression following environmental heat exposure or dehydration. Controlled experimental studies and clinical investigations will therefore be required to validate the proposed molecular framework.

Finally, transcriptomic validation should be interpreted cautiously because transcript abundance does not necessarily correspond to protein abundance. Differences in post-transcriptional regulation, translational efficiency, protein turnover, and post-translational modifications may result in discordance between gene expression and protein expression. Accordingly, the transcriptomic analyses performed in the present study provide complementary biological support for the proposed framework rather than direct validation of the salivary proteomic findings.

Despite these limitations, the present study establishes a novel systems-level framework integrating heat-stress-responsive molecular pathways within the human salivary proteome and provides a foundation for future experimental, longitudinal, and population-based investigations aimed at validating salivary biomarkers of environmental heat exposure and exploring their potential applications in oral biology, environmental health, and occupational medicine.

## 4. Materials and Methods

### 4.1. Human Salivary Proteome Dataset

The primary dataset used in this study was derived from the Human Salivary Proteome Wiki (HSP Wiki)—https://www.salivaryproteome.org/ (accessed on 10 August 2026)—a publicly accessible repository that integrates large-scale proteomic investigations of human saliva. The database was served as the primary source of experimentally identified human salivary proteins. The HSP Wiki compiles proteins identified using high-throughput proteomic technologies, including mass spectrometry-based studies, and represents one of the most comprehensive resources currently available for salivary proteome research. All publicly available human salivary proteomic datasets available in the HSP Wiki at the time of data retrieval were considered eligible for inclusion. Datasets reporting experimentally identified proteins from whole saliva and major salivary gland secretions were included.

Proteomic datasets from multiple large-scale human saliva studies available within the HSP Wiki were retrieved and integrated into a unified master database. Protein accession identifiers and associated protein names were extracted from each dataset and subsequently merged into a single analytical framework. To maximize coverage of the human salivary proteome, datasets derived from whole saliva as well as major salivary gland secretions were included whenever available within the repository. This approach enabled the incorporation of proteins identified under different experimental conditions and biological sampling protocols.

Following data integration, duplicate entries were removed based on protein accession identifiers, generating a non-redundant master salivary proteome dataset. The initial integrated dataset comprised 887,293 protein entries obtained from all included studies. After deduplication, 15,594 unique protein accession identifiers were retained. These accessions corresponded to 2540 distinct protein names and gene products. The difference between the number of accession identifiers and distinct proteins reflects the presence of protein isoforms, alternative database annotations, and multiple accession records associated with the same biological protein entity. Entries lacking valid protein accession identifiers, duplicate accession records, and incomplete protein annotations preventing reliable protein identification were excluded prior to downstream analyses.

The rationale for combining multiple proteomic studies was to reduce potential biases associated with individual experiments, increase proteome coverage, and improve the likelihood of identifying proteins consistently reported across independent salivary proteomic investigations. The resulting master dataset included proteins involved in diverse biological functions, including immune regulation, epithelial protection, stress adaptation, metabolic processes, salivary secretion, and cellular homeostasis, thereby providing a broad representation of the molecular landscape of human saliva.

A summary of the datasets included in the integration process, together with the number of entries and their contribution to the final master salivary proteome, is presented in Table 7. The integrated salivary proteome dataset was subsequently subjected to preprocessing, functional annotation, and heat-stress-related filtering procedures in order to identify proteins potentially involved in heat-stress response, oxidative-stress regulation, inflammatory signaling, water transport, salivary secretion, and mucosal protection.

### 4.2. Data Integration and Preprocessing

The datasets retrieved from the Human Salivary Proteome Wiki were processed through a standardized integration and preprocessing workflow. Data extraction, integration, deduplication, and quality-control procedures were performed using Python version 3.14 (Python Software Foundation, Wilmington, DE, USA), primarily employing the pandas (version 3.0.1) and NumPy (version 2.4.2) libraries.

Initially, all available protein entries were extracted from the selected proteomic studies and merged into a single database. The resulting integrated dataset contained 887,293 protein records derived from multiple large-scale salivary proteomic investigations. Because the same proteins were frequently reported across independent studies, a deduplication procedure was performed using protein accession identifiers as the primary matching criterion. Duplicate accession numbers were removed while preserving the corresponding protein annotation information.

Following deduplication, accession identifiers and protein names were standardized to ensure consistency among datasets originating from different experimental platforms and reporting formats. Quality-control procedures included the identification of incomplete records, removal of entries lacking valid protein accession identifiers, and verification of annotation consistency. Records without sufficient protein identification information were excluded from subsequent analyses.

The resulting master dataset consisted of 15,594 unique protein accession identifiers corresponding to 2540 distinct protein names. To facilitate downstream analyses, a simplified analytical dataset containing accession identifiers and associated protein names was generated and exported for functional annotation, heat-stress-related filtering, protein–protein interaction analysis, and enrichment procedures.

**Figure 7:**
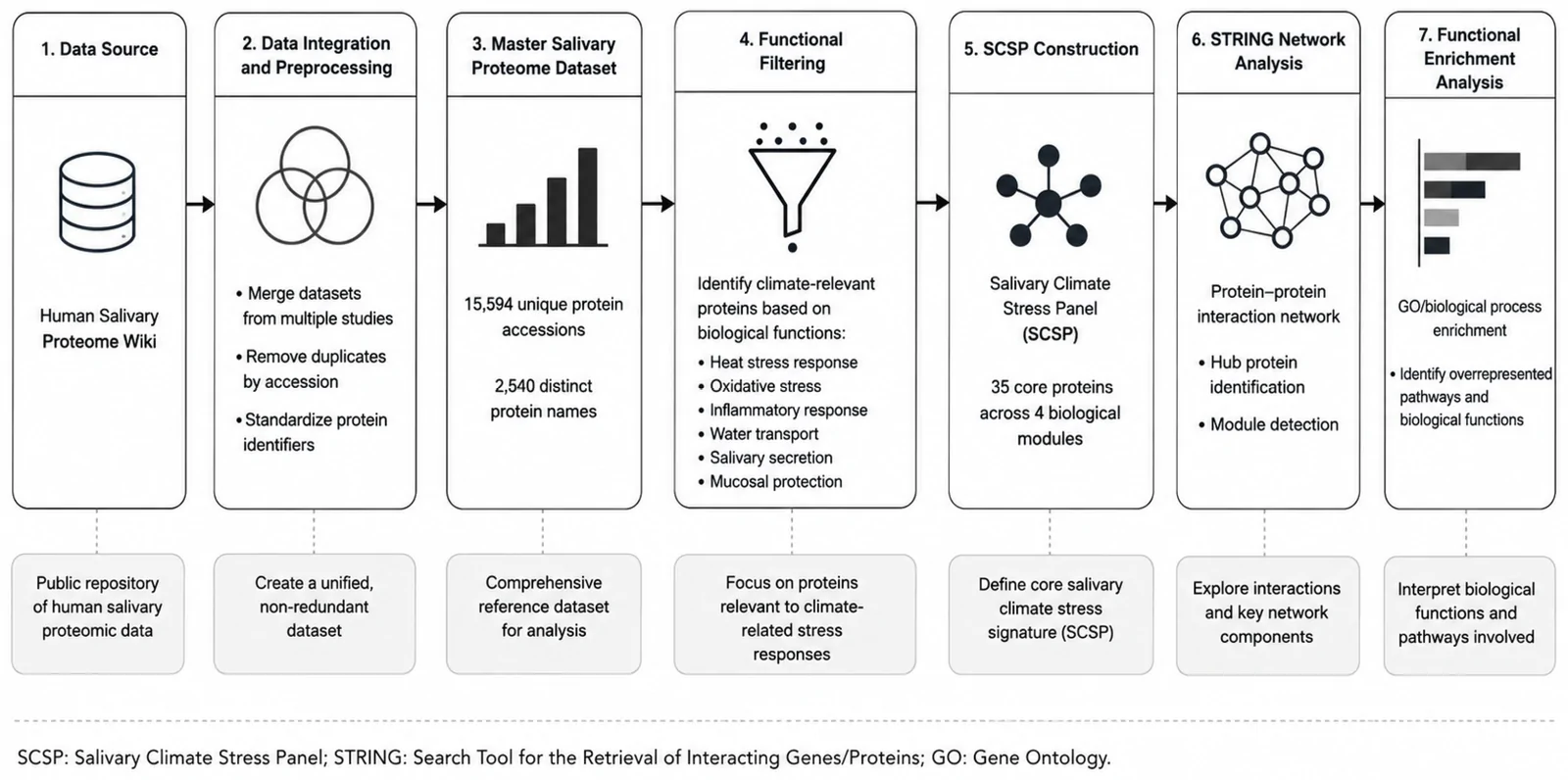
Workflow of the study. Salivary proteomic datasets were retrieved from the Human Salivary Proteome Wiki, integrated into a master salivary proteome dataset, subjected to functional filtering for heat-stress-related proteins, and subsequently analyzed through protein–protein interaction network analysis and functional enrichment procedures to establish the proposed Salivary Climate Stress Panel (SCSP) and the resulting Heat-Stress-Responsive Salivary Signature.

The complete workflow used for dataset integration, preprocessing, heat-stress-related protein identification, network construction, functional enrichment, and external transcriptomic validation is summarized in Figure 7.

### 4.3. Functional Filtering Strategy

To identify salivary proteins potentially involved in heat-stress-related biological responses, a multi-stage functional filtering strategy was applied to the master salivary proteome dataset. The objective of this procedure was to reduce the complexity of the integrated proteome while retaining proteins associated with molecular mechanisms involved in physiological adaptation to environmental heat stress.

Initially, the simplified master salivary proteome dataset was subjected to a custom Python-based computational filtering workflow. Protein names were automatically screened using a predefined panel of biologically relevant keywords representing six functional categories: (i) heat stress response and protein folding, (ii) oxidative stress regulation, (iii) inflammatory signaling and immune response, (iv) water transport and homeostasis, (v) salivary secretion and glandular function, and (vi) mucosal protection and antimicrobial defense. The keyword panel was developed from established biological terminology describing molecular processes involved in heat stress adaptation and salivary physiology.

Representative filtering keywords included *“*heat stress”, “heat shock”, “response to stress”, “oxidative stress”, “reactive oxygen species”, “antioxidant”, “cytokine”, “inflammation”, “water transport”, “water homeostasis”, “salivary secretion”, “osmotic stress”, “mucin”, “lysozyme”, and “lactoferrin”, together with biologically related descriptors. Proteins matching one or more predefined keywords were assigned to one or more functional categories and retained as candidate proteins for further analyses.

Following computational filtering, candidate proteins underwent expert-driven biological curation based on publicly available protein annotations and established biological knowledge. This step confirmed category relevance and distinguished broad keyword matches from proteins with a direct, interpretable role in the proposed framework. The final SCSP was defined before STRING topology and enrichment analyses; node degree and enrichment results were therefore used to characterize the selected panel, not as retrospective inclusion criteria.

Application of the computational filtering workflow reduced the master salivary proteome from 2540 distinct proteins to 2050 candidate proteins associated with one or more predefined functional categories. This filtered subset constituted the candidate pool from which the final SCSP was established through the expert-driven refinement described in Section 4.4.

**Figure 8:**
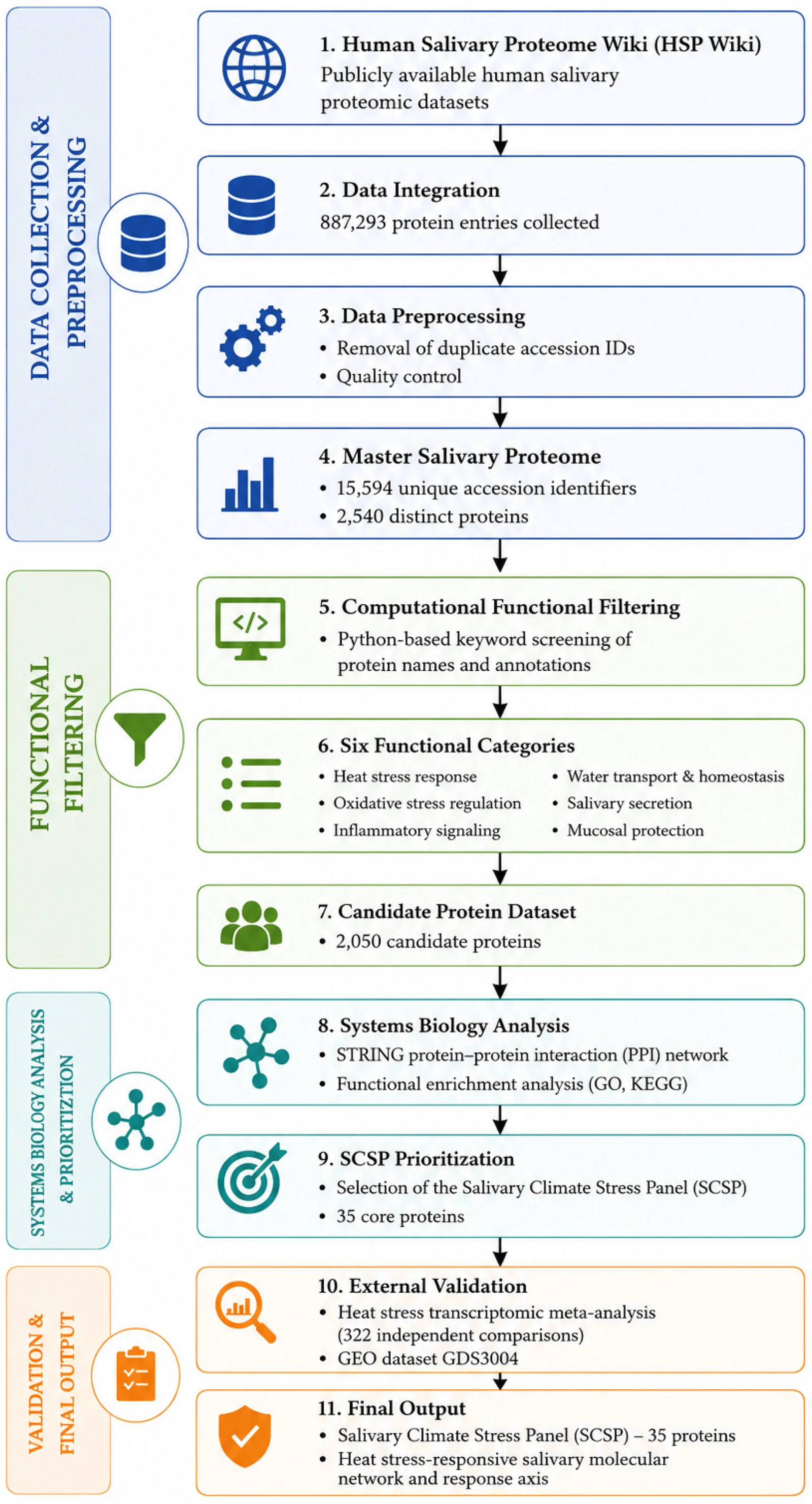
Computational workflow used for the identification and prioritization of the Salivary Climate Stress Panel (SCSP).

The overall functional filtering workflow and the biological categories used for protein selection are summarized in Figure 8 and Table 8, respectively.

### 4.4. The Framework of the Salivary Climate Stress Panel (SCSP)

Following functional filtering, a subset of heat-stress-relevant proteins was identified from the master salivary proteome dataset. To establish a focused and biologically interpretable framework for subsequent network analyses, a Salivary Climate Stress Panel (SCSP) was developed.

The construction of the SCSP was based on the principle that physiological adaptation to environmental heat stress is mediated through a limited number of interconnected biological processes rather than through isolated proteins. Therefore, proteins were selected to represent the principal molecular mechanisms expected to participate in salivary responses to environmental stress.

The expert-driven refinement followed a sequential decision process. First, eligibility required that a protein (i) was present in the non-redundant human salivary proteome and (ii) matched at least one of the six predefined categories through its name or annotation. Second, eligible proteins were prioritized when public annotations and established biological knowledge supported a direct role in heat-shock/protein-folding responses, redox control, inflammatory signaling, water transport, salivary secretion, or mucosal protection. Third, selection favored canonical pathway representatives and proteins with clear salivary or oral relevance. Finally, the panel was reviewed for functional coverage: both regulatory proteins and downstream effectors were retained, while proteins with substantially overlapping roles within the same category were minimized.

No quantitative ranking score, abundance threshold, or network-degree cut-off was used to determine inclusion, because the source repository integrates heterogeneous studies and does not provide directly comparable abundance estimates across all proteins. The final size of 35 was therefore not statistically optimized. It represents a purposive expert-curated subset designed to preserve all six predefined domains while remaining interpretable for network analysis. Consequently, the SCSP should be understood as a transparent hypothesis-generating panel rather than an exhaustive or uniquely determined set of heat-responsive salivary proteins.

The final SCSP consisted of 35 proteins distributed across six biological categories. The panel included heat shock proteins (HSPA1A, HSPA8, HSP90AA1, HSP90AB1, DNAJB1), antioxidant and redox regulators (SOD1, SOD2, CAT, TXN, PRDX1, PRDX2, GPX1), inflammatory mediators (IL6, TNF, IL1B, CXCL8, NFKB1, STAT3, PTGS2), water transport and homeostasis proteins (AQP1, AQP5, CFTR, ATP1A1, SLC12A2), salivary secretion-associated proteins (AMY1A, CA6, CST1, CST3, DMBT1), and mucosal protection proteins (MUC5B, MUC7, LTF, LYZ, BPIFA2, DEFA1).

A complete overview of the proteins included in the proposed Salivary Climate Stress Panel and their functional classification is presented in Table 9. The SCSP was subsequently used as the input dataset for protein–protein interaction network analysis and functional enrichment procedures. By integrating proteins representing complementary biological functions, the proposed panel was designed to capture the molecular architecture of salivary adaptation to heat-stress-related stressors.

### 4.5. STRING Protein–Protein Interaction Network Analysis

To investigate the interactions among proteins included in the Salivary Climate Stress Panel (SCSP), a protein–protein interaction (PPI) network analysis was performed using the STRING database (Search Tool for the Retrieval of Interacting Genes/Proteins, version 12.0). STRING is a widely used systems biology platform that integrates experimentally validated and computationally predicted protein interactions from multiple biological sources.

The 35 proteins comprising the SCSP were imported into STRING using *Homo sapiens* (Taxonomy ID: 9606) as the reference organism. Network construction was performed using a high-confidence interaction score threshold of 0.700 in order to prioritize biologically reliable associations.

Interaction sources included experimentally validated interactions, curated pathway databases, co-expression evidence, and knowledge-based associations available within the STRING platform. Disconnected nodes were excluded from graphical visualization but retained for downstream functional interpretation.

The resulting network was used to evaluate the overall connectivity of the proposed panel and to identify highly connected proteins potentially acting as central regulators of heat-stress-responsive biological processes. Standard network topology metrics provided by STRING were examined, including the number of nodes, number of edges, average node degree, local clustering coefficient, and protein–protein interaction enrichment statistics.

In addition, protein node degree values were extracted to identify hub proteins within the network. Proteins exhibiting the highest degree values were considered candidate hub proteins and potential regulators of network organization. Particular attention was given to proteins linking heat stress, oxidative stress, inflammatory signaling, and water homeostasis modules, as these interactions were hypothesized to represent the molecular core of the Salivary Heat Stress Response.

### 4.6. Functional Annotation and Enrichment Analysis

Functional annotation and enrichment analyses were performed to characterize the biological processes represented within the Salivary Climate Stress Panel (SCSP) and to evaluate the functional coherence of the proposed heat-stress-responsive protein network.

Functional information associated with SCSP proteins was obtained from publicly available biological annotation resources integrated within the STRING platform (version 12.0). Biological processes, molecular functions, and cellular pathways associated with each protein were examined to identify mechanisms potentially involved in physiological adaptation to environmental heat stress. Particular attention was given to biological processes associated with heat-stress response, oxidative-stress regulation, inflammatory signaling, water transport, osmotic regulation, salivary secretion, and cellular homeostasis. Functional annotations were reviewed to determine whether the proteins included in the SCSP converged toward common biological pathways and stress-response mechanisms.

To further evaluate the biological relevance of the identified protein network, enrichment analyses were performed using the functional annotation framework implemented within STRING. Overrepresented biological processes were identified by comparing the frequency of protein participation within specific functional categories against the expected distribution in the Homo sapiens reference proteome. Statistical significance was assessed using the enrichment algorithms provided by STRING, and multiple testing correction was performed using the Benjamini–Hochberg false discovery rate (FDR) procedure. Biological processes with an FDR-adjusted *p*-value < 0.05 were considered significantly enriched and retained for interpretation. Functional categories associated with environmental stress adaptation were subsequently examined and interpreted in the context of salivary physiology and oral homeostasis.

The resulting enrichment profiles were used to identify the principal biological processes represented within the SCSP and to support the development of a system-level model describing heat-stress-responsive pathways in human saliva.

### 4.7. External Validation Using Public Heat-Stress Transcriptomic Datasets

To provide independent validation of the proposed SCSP, publicly available human heat-stress transcriptomic datasets were analyzed. Validation was performed using a publicly available large-scale human heat-stress transcriptomic meta-analysis integrating 322 independent heat-stress versus control transcriptomic comparisons retrieved from the Gene Expression Omnibus (GEO) database and other public repositories.

The meta-analysis was performed by Yonezawa and Bono and reported 322 human heat-stress versus non-treatment comparisons [35]. It generated Heat-Stress–Non-treatment (HN) scores for individual genes, calculated as the number of upregulated comparisons minus the number of downregulated comparisons. Positive HN scores therefore indicate recurrent upregulation under heat-stress conditions, whereas negative scores indicate recurrent downregulation [35].

Genes included in the SCSP were matched against the HN-score dataset to evaluate whether proteins identified through salivary proteome network analysis overlapped with established heat-stress-responsive molecular pathways. Particular attention was given to the principal hub proteins identified through STRING network analysis, including HSPA1A, HSPA8, HSP90AA1, DNAJB1, TNF, and AQP1, which were examined for concordant transcriptomic responses under heat-stress conditions.

In addition, the publicly available GEO dataset GDS3004 (Heat Stress Effect on Human Epithelial Cells) was analyzed as an independent transcriptomic model of cellular thermal stress. Expression patterns of representative SCSP genes were examined to verify their transcriptional responses under controlled heat-stress conditions and to provide an independent assessment of the biological relevance of the proposed protein network.

The combined validation strategy enabled assessment of whether the molecular modules identified within the proposed Heat-Stress-Responsive Salivary Signature corresponded to canonical heat-stress-responsive pathways consistently observed across independent human transcriptomic studies. Custom Python scripts were used to perform data integration, preprocessing, and functional filtering according to the workflow described in this study.

## 5. Conclusions

This study represents, to our knowledge, the first systems-level investigation of heat-stress-responsive molecular pathways within the human salivary proteome. By integrating large-scale salivary proteomic datasets with protein–protein interaction network analysis, Gene Ontology functional annotation, and independent transcriptomic validation, we developed the Salivary Climate Stress Panel (SCSP) and proposed the Salivary Heat Stress Response Axis, a conceptual framework integrating heat stress response, oxidative stress regulation, inflammatory signaling, and water homeostasis.

Protein–protein interaction analysis identified heat shock proteins as the principal molecular hubs within the proposed framework, whereas Gene Ontology functional annotation highlighted water transport and salivary secretion as the predominant biological processes represented within the SCSP. Together, these findings support the concept that salivary adaptation to environmental heat stress involves coordinated interactions among stress-response, antioxidant, inflammatory, and homeostatic pathways rather than isolated molecular mechanisms.

Although these analyses do not establish causality or confirm heat-induced changes in salivary protein abundance, they provide a biologically plausible framework for experimental testing. The proposed SCSP and Salivary Heat Stress Response Axis should next be evaluated in prospective in vivo studies that collect saliva from people exposed to defined heat conditions, ideally before, during, and after exposure. Targeted proteomic measurements should be combined with exposure intensity and duration, hydration status, salivary flow, xerostomia symptoms, periodontal indices, and oral mucosal examination.

Priority populations include outdoor and industrial workers, athletes, military personnel, and communities exposed to recurrent extreme temperatures. Longitudinal sampling across acute and repeated exposures will be needed to determine whether the SCSP is specific to heat, distinguishes heat from dehydration or physical exertion, and predicts clinically relevant oral outcomes. Only after such validation should individual SCSP proteins or combinations be considered candidate biomarkers for environmental or occupational monitoring.

## Figures and Tables

**Table 1:** Summary of the functional filtering process.

Parameter	Value
Climate-relevant candidate proteins	2050
Heat stress proteins	321
Oxidative stress/ROS proteins	272
Water transport/dehydration proteins	111
Inflammation/immune response proteins	747
Mucosal protection proteins	482
Final SCSP proteins	35

**Table 2:** Proposed Salivary Climate Stress Panel (SCSP).

Category	Number of Proteins	Proteins
Water Transport	5	AQP5, AQP1, CFTR, ATP1A1, SLC12A2
Heat Stress	5	HSPA1A, HSPA8, HSP90AA1, HSP90AB1, DNAJB1
Oxidative Stress	7	SOD1, SOD2, GPX1, PRDX1, PRDX2, CAT, TXN
Inflammation	7	IL6, TNF, IL1B, CXCL8, NFKB1, STAT3, PTGS2
Mucosal Protection	6	MUC5B, MUC7, LTF, LYZ, BPIFA2, DEFA1
Salivary Homeostasis	5	AMY1A, CA6, CST1, CST3, DMBT1
Total	35	Salivary Climate Stress Panel (SCSP)

**Table 3:** General characteristics of the SCSP protein–protein interaction network.

Network Property	Result
SCSP input identifiers	35
Mapped STRING nodes	34
Protein–protein associations (edges)	72
Average node degree	4.24
Average local clustering coefficient	0.618
Expected number of edges	13
PPI enrichment *p*-value	<1.0 × 10^−16^
Major functional modules	4
Largest functional module	Heat stress response
Principal hub proteins	HSP90AB1, HSP90AA1, IL1B
Dominant biological organization	Heat stress, inflammation, oxidative stress, and water homeostasis

**Table 4:** Top hub proteins identified within the SCSP network.

Rank	Protein	Degree	Functional Category
1	HSP90AB1	12	Heat Stress
2	HSP90AA1	11	Heat Stress
3	IL1B	9	Inflammation
4	IL6	8	Inflammation
5	NFKB1	8	Inflammation
6	SOD1	8	Oxidative Stress
7	STAT3	8	Inflammation
8	HSPA8	7	Heat Stress
9	PRDX1	7	Oxidative Stress
10	TXN	7	Oxidative Stress

**Table 5:** Representative significantly enriched Gene Ontology biological processes associated with the SCSP (STRING version 12.0; Benjamini–Hochberg’s FDR < 0.05).

GO ID	Biological Process	Gene Count	FDR-Adjusted *p*-Value	Matching SCSP Proteins
GO:0006970	Response to osmotic stress	7	7.46 × 10^−8^	SLC12A2, AQP5, AQP1, HSP90AA1, PTGS2, HSP90AB1, TNF
GO:0034614	Cellular response to reactive oxygen species	7	7.63 × 10^−7^	CAT, PRDX1, SOD1, PRDX2, AQP1, IL6, SOD2
GO:0034599	Cellular response to oxidative stress	8	1.58 × 10^−6^	CAT, PRDX1, SOD1, PRDX2, AQP1, HSPA1A, IL6, SOD2
GO:0006979	Response to oxidative stress	9	3.37 × 10^−6^	CAT, PRDX1, SOD1, PRDX2, AQP1, PTGS2, HSPA1A, IL6, SOD2
GO:0051092	Positive regulation of NF-κB transcription factor activity	6	5.48 × 10^−5^	LTF, CAT, IL1B, STAT3, HSPA1A, TNF
GO:0009266	Response to temperature stimulus	6	8.22 × 10^−5^	SOD1, HSP90AA1, PTGS2, HSP90AB1, HSPA1A, SOD2
GO:0009408	Response to heat	5	1.20 × 10^−4^	SOD1, HSP90AA1, PTGS2, HSP90AB1, HSPA1A
GO:0061844	Antimicrobial humoral immune response mediated by antimicrobial peptide	5	2.00 × 10^−4^	LTF, CXCL8, DMBT1, DEFA1, MUC7
GO:0034605	Cellular response to heat	4	2.20 × 10^−4^	HSP90AA1, PTGS2, HSP90AB1, HSPA1A
GO:0006986	Response to unfolded protein	5	2.70 × 10^−4^	DNAJB1, HSP90AA1, HSP90AB1, HSPA1A, HSPA8

**Table 6:** External validation of representative SCSP genes using the human heat-stress meta-analysis database.

Gene	Functional Module	HN Score
HSPA1A	Heat Stress	239
DNAJB1	Heat Stress	210
TNF	Inflammation	68
HSP90AA1	Heat Stress	68
HSPA8	Heat Stress	47
HSP90AB1	Heat Stress	20
AQP1	Water Homeostasis	17
GPX1	Oxidative Stress	10
SOD1	Oxidative Stress	9
IL6	Inflammation	9
TXN	Oxidative Stress	7
CAT	Oxidative Stress	6
NFKB1	Inflammation	6

**Table 7:** Summary of the human salivary proteome datasets included in the study.

Dataset	Source	Initial Entries	Role in Analysis
Dataset 1	Human Salivary Proteome Wiki	295	Historical reference dataset
Dataset 2	Human Salivary Proteome Wiki	129,150	Large-scale proteomic dataset
Dataset 3	Human Salivary Proteome Wiki	757,848	Primary proteomic dataset
Integrated Dataset	Combined datasets	887,293	Master salivary proteome
Unique Protein Accessions	After deduplication	15,594	Final analytical dataset
Distinct Protein Names	After deduplication	2540	Functional analysis dataset

**Table 8:** Functional categories and representative biological processes used for protein selection during the functional filtering strategy.

Functional Category	Representative Biological Processes	Example Proteins
Heat Stress Response	Protein folding, cellular stress response, heat shock response	HSPA1A, HSPA8, HSP90AA1
Oxidative Stress Regulation	ROS detoxification, antioxidant defense, redox regulation	SOD1, SOD2, CAT, TXN
Inflammatory Signaling	Cytokine signaling, immune response, inflammation	IL6, TNF, IL1B, NFKB1
Water Homeostasis	Water transport, osmotic regulation, fluid balance	AQP1, AQP5, CFTR
Salivary Secretion	Glandular secretion, salivary physiology	AQP5, CA6, AMY1A
Mucosal Protection	Antimicrobial defense, epithelial protection	MUC5B, MUC7, LTF, LYZ

**Table 9:** The proteins included in the SCSP and their functional classification.

Category	Number of Proteins	Representative Proteins
Heat Stress Response	5	HSPA1A, HSPA8, HSP90AA1, HSP90AB1, DNAJB1
Oxidative Stress Regulation	7	SOD1, SOD2, CAT, TXN, PRDX1, PRDX2, GPX1
Inflammatory Signaling	7	IL6, TNF, IL1B, CXCL8, NFKB1, STAT3, PTGS2
Water Homeostasis	5	AQP1, AQP5, CFTR, ATP1A1, SLC12A2
Salivary Secretion	5	AMY1A, CA6, CST1, CST3, DMBT1
Mucosal Protection	6	MUC5B, MUC7, LTF, LYZ, BPIFA2, DEFA1
Total	35	SCSP

## Data Availability

The salivary proteome data used in this study are publicly available from the Human Salivary Proteome Wiki (HSP Wiki; https://www.salivaryproteome.org/; accessed on 10 August 2026). The external heat-stress meta-analysis is described in Yonezawa and Bono [35].

## Abbreviations

The following abbreviations are used in this manuscript:

AQP	Aquaporin
CAT	Catalase
CFTR	Cystic Fibrosis Transmembrane Conductance Regulator
CXCL8	C-X-C Motif Chemokine Ligand 8 (Interleukin-8)
DNAJB1	DnaJ Heat Shock Protein Family (Hsp40) Member B1
FDR	False Discovery Rate
GEO	Gene Expression Omnibus
GO	Gene Ontology
GPX1	Glutathione Peroxidase 1
HN Score	Heat-Stress–Non-treatment Score
HSP	Heat Shock Protein
HSP Wiki	Human Salivary Proteome Wiki
IL1B	Interleukin 1 Beta
IL6	Interleukin 6
NF-κB	Nuclear Factor Kappa B
NFKB1	Nuclear Factor Kappa B Subunit 1
PPI	Protein–Protein Interaction
PRDX	Peroxiredoxin
ROS	Reactive Oxygen Species
SCSP	Salivary Climate Stress Panel
SOD	Superoxide Dismutase
STAT3	Signal Transducer and Activator of Transcription 3
STRING	Search Tool for the Retrieval of Interacting Genes/Proteins
TNF	Tumor Necrosis Factor
TXN	Thioredoxin
